# Relationships between physical function, body composition and metabolic health in Pacific Island youth

**DOI:** 10.1371/journal.pone.0260203

**Published:** 2022-02-17

**Authors:** Elaine C. Rush, Tara Coppinger, Shabnam Jalili-Moghaddam, El-Shadan Tautolo, Lindsay D. Plank

**Affiliations:** 1 School of Sport and Recreation, Auckland University of Technology, Auckland, New Zealand; 2 Centre for Pacific Health and Development Research, Auckland University of Technology, Auckland, New Zealand; 3 Department of Sport, Leisure & Childhood Studies, Munster Technological University, Cork, Ireland; 4 Department of Surgery, University of Auckland, Auckland, New Zealand; Universidad Pablo de Olavide, SPAIN

## Abstract

The Pacific Islands Families (PIF) study is a birth cohort study designed to increase knowledge about the growth and development of Pacific children living in Auckland, New Zealand. Adolescence is a critical time of growth and development, yet the roles of physical function and body composition in metabolic health at this life stage are not clear. We aimed to investigate associations between measures of physical function (the 6-minute-walk-test (6MWT)), heart rate changes before and after the 6MWT, handgrip strength, body composition including appendicular skeletal muscle mass (ASMM) measured by dual-energy X-ray absorptiometry and biomarkers of metabolic health from a fasting blood sample.A total of 200 youth (98 girls, 102 boys) aged 14–15 years, from the birth-cohort of children in the Pacific Islands families study were measured. In girls, the proportion of ASMM was lower and fat higher than in boys. Controlling for age, a 1% increase in ASMM predicted a longer walk distance (+6.3, 95%CI 2.2, 10.4 m in girls; +7.1, 95%CI 4.4, 9.1 m in boys) and lower heart rate following the 6MWT. ASMM and fat mass were independently predictive of maximal handgrip strength which was increased by 1.4 (1.0,1.8) kg in girls and 1.7 (1.3, 2.0) kg in boys for each kg increase in ASMM and reduced by 0.23 (0.08, 0.38) kg in girls and 0.26 (0.14, 0.37) kg in boys for each kg increase in fat mass. Lower total cholesterol and LDL were associated with an increase in distance walked in boys only. For each year of age, distance walked was reduced by 34 (15, 53) m in girls and 59 (36,84) m in boys. These findings should be explored further in the context of other influences such as food security, opportunities for physical activity and cultural expectations.

## Introduction

Across the lifecourse, physical function, defined as the capacity to undertake physical activities, is an important marker of health [1,2]. Although physical function and fitness is, in part, genetically determined, nutritional status and environmental factors also play a role [3]. Timely, accurate measures of physical fitness are subsequently important but, to date, there remains limited evidence from prospective studies for lifecourse pathways for a protective effect of physical fitness and competence on metabolic health [4]. Systematic reviews and meta-analyses of longitudinal studies have examined the association of muscular fitness [5] and cardiorespiratory fitness [6] of children and adolescents with follow-up measures of health between 1 and 30 years and found improved fitness predicted better future health. In adolescence the relationship of physical function and body composition to current markers of metabolic health is not clear.

Measurement of physical function is complex [2] since no single criterion measure is recognised as the gold standard. Pragmatically, to maximize compliance, assessments should be simple to administer, require minimal equipment and be easily managed over a short time. Understanding the determinants of physical function is enhanced if skeletal muscle mass and fat mass are considered [7] and if they are assessed across a range of body sizes. The use of universal BMI cut-off points may not be appropriate for the comparison of fatness and muscularity among ethnic groups [8], plus variations in maturation and height must be considered in field-based measures of physical function including physical fitness [9].

Pacific Island people are amongst the most muscular individuals in the world. When compared with other ethnic groups, Pacific Island adults [8] and children [10] have more muscle and less fat for the same height and weight. They also traditionally have a larger body frame and more central than peripheral fat, which could represent adaptations that occurred before and during migration across the Pacific Ocean [11].

The Pacific Island population in New Zealand comprises 8.1% of the total population with a median age of 23 years [12]. Consistently over the last two decades, more than 50% of Pacific Island children and 85% of Pacific Island adults were identified as either overweight or obese [13]. It is known that the prevalence of overweight and obesity increases with age and those living in more socioeconomically deprived areas are more likely to have excess weight [13] which is associated with chronic diseases. Pacific peoples experience more chronic disease than other New Zealanders [13]. Chronic diseases associated with poorer health include cardiovascular and cerebrovascular diseases, cancer and type 2 diabetes mellitus

In the year 2000, a unique longitudinal study entitled the Pacific Islands Families (PIF) study was initiated in order to gain an insight into the growth, health, psychosocial and behavioural characteristics of Pacific Island children born in New Zealand. Briefly, mothers of Pacific infants born at Middlemore Hospital (Auckland, New Zealand) between 15 March and 17 December 2000 were recruited. Maternal home interviews covering socio-demographic, cultural, environmental, child development, family and household dynamics, childcare, lifestyle, and health issues were undertaken at approximately 6 weeks and 1, 2, 4, 6, 9 and 14 years postpartum. Findings from the cohort [14] showed that the children of the PIF Study gained weight more rapidly from birth [15], and matured earlier than children of other ethnicities [16]. More than 40% were obese and a further 30% were overweight by the age of 14 years [17]. In this cohort, we have also found that children with slower weight gain between 2.5 to 13.5 years or lower body weights in early life exhibited better metabolic health parameters [18].

The PIF study presents a unique opportunity to examine the association of physical function with body composition and metabolic health at the age of 14 years (a critical period of growth and development). Consequently, the aim of this analysis was to examine associations between distance walked in 6 minutes, heart rate changes and handgrip strength in relation to body composition and biomarkers of metabolic health in Pacific Island youth. Specifically, it was hypothesised that appendicular skeletal muscle mass (ASMM) would be a strong and positive predictor of physical function and metabolic health.

## Method

This is a cross-sectional analysis of a longitudinal cohort study of participants born in the year 2000. In 2014, 931 (66%) of the original cohort had field measures of height and weight recorded. A nested subsample (n = 204) was drawn by randomly selecting 10 males and 10 females from each decile of body weight measured at age 11 years. The profile of the cohort and the nested substudy at 14–15 years has been reported previously [17]. In order to assess pubertal status youth were asked questions about experiencing a growth spurt in height, pubic hair and skin changes, facial hair growth in boys and the development of breasts and menarche for girls [19]. The responses to these questions were coded as present or not present. These same questions were asked of parents when the child was 9 and 11 years of age [16].

Substudy participants attended the Body Composition Laboratory at the University of Auckland after overnight fasting. Height (Seca 206, Hamburg, Germany) and weight (Seca 703) were measured in light clothing. Body mass index (BMI) was calculated as weight in kg divided by height in metres squared. Age, in years, was determined from date of measurement minus date of birth. The 204 participants undertook a 6-minute walk test (6MWT) with measures of heart rate (Onyx 9550, Nonin, MN, USA) at 0, 6 and 7 min.

The 6MWT was conducted according to standardized protocols [20]. Participants were told that ‘the purpose of this test is to see how far you can walk in 6 minutes’. Youth were shown how to walk and turn quickly at the ends of the course, which was marked at the start and at 20 metres. Each participant received standardized individual encouragement throughout. Laps were recorded with a lap counter, and partial lap distance estimated from markers which were placed along the course at 5 m intervals. Duration of the test was timed with a stopwatch. Pedometers (Yamax Digi-Walker SW-200 Yamasa Tokei Keiki Co. Ltd, Japan) were worn by participants and the number of steps noted as a quality control check of the distance recorded.

Grip strength of the dominant hand was measured by asking the participants to grip the hand dynamometer as tightly as possible (Jamar Plus, Mentone Educational Centre, Melbourne, Australia). The maximum of three measurements was recorded. Body composition was measured by dual-energy X-ray absorptiometry (iDXA, GE Healthcare, Madison, WI) and the system software (version 13) provided the total mass, fat, lean soft tissue, and bone mineral content of the whole body and by regional analysis the limbs (legs plus arms). Percent body fat was calculated as 100xtotal body fat mass/total mass. ASMM was derived from the DXA scans as total limb mass minus the sum of limb fat and wet bone mass, estimated as bone mineral content divided by 0.55 [21]. Percent ASMM (%ASMM) was calculated as 100xASMM/total mass.

As previously published [18] the growth trajectory in 1053 children from the PIF study cohort was determined from z scores calculated at each of the ages 2.5, 4, 6, 9, 11,and 13.5 years. For each child the slope and intercept from the linear regression (weight = slope x age+intercept) were determined to describe the z score linear trend intercept and slope.

Venous blood samples were collected for biomarkers of metabolic health. Samples for glucose, lipid profile, 25(OH) vitamin D, and high sensitivity C‐reactive protein (CRP) were sent to the hospital‐accredited laboratory (LabPLUS) for same‐day processing using standard automated procedures (Cobas C8000 modular analyser, Roche Diagnostics) with photometric and electrochemiluminescence detection methods. Remaining samples were centrifuged immediately at 4°C and plasma stored at −80°C for later batch processing. Insulin and C‐peptide were measured using an electrochemiluminescence immunoassay (Cobas e411, Roche Diagnostics). Insulin resistance was calculated with the homeostatic model assessment (HOMA2IR) computer program from fasting glucose and insulin [22]. The presence of metabolic syndrome was identified as abdominal obesity plus two or more clinical features defined according to the International Diabetes Federation criteria for ages 10 to 16 [23]. Abdominal obesity was defined as a waist circumference greater than the sex and age specific 90^th^ percentile thresholds [24].

Ethics approval for the nested substudy was obtained from the Central Health and Disability Ethics Committee (ref. 8/CEN/108). Parents provided written consent and the youth assent for the study.

### Statistics

Statistical analyses were carried out using SPSS (version 25, IBM corporation). Data were checked for normality by visual observation of histograms and quantile-quantile plots. Skewed data (CRP) were transformed (natural logarithm) for analysis. CRP concentrations reported as <1 mg/L were taken as 1.0 mg/L. A Winsorization approach was adopted for 4 outliers with CRP>10 mg/L. Data are presented as mean and SD with 95% confidence intervals for sex differences, tested by Independent t test. Correlation, linear and stepwise multiple regression analyses were conducted to investigate predictors of 6-min walk distance, heart rate and handgrip strength.

## Results

Physical and metabolic characteristics of 200 youth (98 girls, 102 boys) who had a satisfactory measure of distance walked in 6 minutes are reported in Table 1. Pubic hair growth was reported by 98.9% and 97.8% of the girls and boys and 97.7% of the girls reported that they had experienced menarche. Girls were shorter (-9 cm), weighed less (-5 kg) and had a lower percentage ASMM (-5%) and higher percent body fat (+9%) than boys (Table 1**)**. Metabolic syndrome was identified in 13 youth, 5 girls and 8 boys. Resting heart rate was not different by sex. In the 6MWT, girls walked 43 m (SE ±8m) less and at 6 minutes reached a higher heart rate (+10 bpm) than boys. Heart rate change in the first minute of recovery was greater in girls and so was their high density lipoprotein (HDL) cholesterol concentration (Table 1).

**Table 1:** Physical and metabolic characteristics of 200 girls and boys.

	Girls N = 98 Mean	SD	Boys N = 102 Mean	SD	Mean difference (95% CI)	P*
**Age, y**	14.9	0.5	14.9	0.4	0.1 (-0.1, 0.2)	0.442
**Weight, kg**	81.1	20.7	85.8	25.2	-4.6 (-11.1,1.8)	0.158
**Height, cm**	166.6	5.6	175.2	7.1	-8.6(-10.4, -6.6)	<0.001
**BMI, kg/m^2^**	29.1	6.6	27.8	7.51	1.3 (-0.7, 3.3)	0.202
**Fat, kg**	31.2	13.1	26.0	15.7	5.2 (1.1, 9.2)	0.012
**Fat, %**	37.2	6.6	28.1	9.4	9.1 (6.9, 11.4)	<0.001
**ASMM, kg**	21.1	4.6	26.7	5.8	-5.4 (-6.9, -4.0)	<0.001
**ASMM, %**	26.4	2.19	31.6	3.6	-5.3 (-6.1, -4.4)	<0.001
**Growth trajectory**						
**Intercept z score**	-0.078	1.286	0.362	1.192	-0.439 (-0.786, -0.094)	0.013
**Slope z score**	0.004	0.105	-0.032	0.088	0.036 (0.009, 0.064)	0.008
**6 min Walk, m**	557.7	48.5	600.5	62.2	-42.8 (-58.4, -27.3)	<0.001
**HR, 0 min**	71.8	10.7	69.0	11.9	2.8 (-0.3, 6.0)	0.078
**HR, 6min**	130.0	21.4	120.9	23.2	9.1 (2.9, 15.3)	0.004
**HR, Δ 0–6 min**	58.2	19.2	51.9	21.8	6.3 (0.6, 12.0)	0.032
**HR, Δ 6–7 min**	19.9	9.5	15.9	12.3	3.9 (0.8, 7.0)	0.013
**Handgrip, kg**	34.2	6.8	42.5	9.6	-8.3 (-10.6, -6.0)	<0.001
**Chol, mmol/L**	3.9	0.5	4.0	0.8	0.0 (-0.2, 2.0)	0.777
**LDL, mmol/L**	2.1	0.5	2.2	0.7	-0.1 (-0.2, 0.1)	0.374
**HDL, mmol/L**	1.4	0.3	1.3	0.3	0.1 (0, 0.2)	0.017
**TG, mmol/L**	0.98	0.40	1.07	0.51	-0.09 (-0.21, 0.04)	0.194
**Insulin, mU/L**	28.3	16.9	27.3	19.0	1.1 (-4.1, 6.2)	0.686
**Insulin resistance**	0.66	0.14	0.70	0.16	-0.03 (0.02, -0.07)	0.168
**C-peptide, ng/mL**	3.5	1.1	3.3	1.3	0.2 (-0.2, 0.5)	0.353
**Vit. D nmol/L**	45.4	18.1	50.7	20.3	-5.2 (-10.6, 0.2)	0.057
**lnCRP**	0.74	0.40	0.74	0.32	0.0 (-0.1, 0.1)	0.996

*Independent t test. ASMM, appendicular skeletal muscle mass; Chol, total cholesterol; HDL, high density lipoprotein; HR, heart rate; LDL, low density lipoprotein; LnCRP, natural logarithm of C-reactive protein; TG, triglycerides. Bolded values are significantly different by sex (p<0.05).

For every increasing year of age, although resting heart rate was not associated with age for girls and boys, mean heart rate at 6 minutes was negatively associated with age for girls (r = -0.31, p = 0.002) and boys (r = -0.34 p<0.001).

The z score for the slope of the growth trajectory to age 14 years was negatively associated with %ASMM for girls (r = -0.25, p = 0.012) and boys (r = -0.34, p<0.0001). No association was seen between the z score for the intercept and %ASMM for girls (r = -0.14, p = 178) but for boys the intercept was negatively associated with %ASMM (r = -0.34, p<0.0001). Percentage body fat was positively associated with both the intercept and the slope z scores for girls (r = 0.26, p = 0.01; r = 0.34, p = 0.001, respectively) and boys (r = 0.41, p<0.0001; r = 0.40, p<0.0001, respectively). In other words, the faster growing children had proportionally less muscle and those with early and fast growth had proportionally more fat.

Age-adjusted linear regression analyses performed for girls (Table 2) and boys (Table 3) separately. **Controlling for age,** for each kg increment in body weight, the distance walked by boys was shorter (Table 3) and the distance walked by girls (Table 2) was unchanged, while heart rate change during the walk test was higher for both groups. Handgrip strength was positively associated with body weight, height and BMI for both sexes. **The higher the percentage fat,** the less distance walked and the higher the heart rate at 6 minutes for both sexes, but there was no significant association of percentage fat with handgrip strength. Each 1% increase in body fat predicted 1.6–2.8 m less distance walked. **The percentage of ASMM positively predicted** a longer walk distance and lower heart rate during exercise for both sexes. A 1% increase in ASMM predicted 6–7 m additional distance. More rapid growth in boys (slope z score) was associated with greater handgrip strength (Table 3) but the same effect was not seen in girls (Table 2). Higher cholesterol and LDL were associated with reduced distance walked in boys with no association seen in girls. In both boys and girls, higher vitamin D predicted a lower heart rate both at rest and following exercise and, in boys only, an increased walk distance.

**Table 2:** Predictors of physical function measures for 98 girls by linear regression adjusted for age.

Predictors	6min Walk, m	P	Heart rate 0 min, bpm	P	Heart rate 6min, bpm	P	Heart rate Δ 0–6 min bpm		Heart rate Δ 6- 7min bpm	P	Handgrip strength, kg	P
**Anthropometric**												
**Weight, kg**	-0.2 (-0.7, 0.2)	0.333	0.1 (-0.0, 0.2)	0.098	**0.3 (0.1, 0.5)**	**0.005**	**0.2 (0.0, 0.4)**	**0.036**	0.0 (-0.1, 0.1)	0.396	**0.1 (0.1, 0.2)**	**<0.0001**
**Height, cm**	1.3 (-0.4, 2.9)	0.126	0.2 (-0.2, 0.6)	0.242	0.5 (-0.2, 1.2)	0.173	0.3 (-0.4, 0.9)	0.405	0.1 (-0.2, 0.5)	0.454	**0.4 (0.1, 0.6)**	**0.002**
**BMI, kg/m^2^**	-1.1 (-3.4, 0.3)	0.132	0.2 (-0.1, 0.6)	0.151	**0.8 (0.2, 1.4)**	**0.007**	**0.6 (0.0, 1.2)**	**0.034**	0.1 (-0.2, 0.4)	0.404	**0.4 (0.2, 0.6)**	**<0.0001**
**Fat, kg**	-0.6 (-1.3, 0.1)	0.095	**0.2 (0.0, 0.3)**	**0.048**	**0.5 (0.2, 0.8)**	**0.003**	**0.3 (0.0, 0.6)**	**0.031**	0.1 (-0.1, 0.2)	0.467	**0.2 (0.1, 0.3)**	**0.001**
**Fat, %**	**-1.6 (-2.5, -0.2)**	**0.027**	**0.4 (0.0, 0.7)**	**0.029**	**1.0 (0.4, 1.6)**	**0.001**	**0.6 (0.1, 1.2)**	**0.023**	0.1 (-0.2, 0.4)	0.405	0.2 (-0.0, 0.4)	0.069
**ASMM, kg**	-0.0 (-2.0, 2.0)	0.927	0.0 (0.0, 0.0)	0.338	1.0 (0.1, 2.0)	0.026	0.8 (-0.0,1.6)	0.056	0.0 (-0.0,0.6)	0.266	**0.8 (0.6, 1.0)**	**<0.0001**
**ASMM, %**	**6.3 (2.2, 10.4)**	**0.003**	**-1.1 (-2.1,-0.2)**	**0.023**	**-2.3 (-4.1, -0.5)**	**0.014**	-1.2(-2.9, 0.5)	0.500	-0.1 (-0.7, 1.0)	0.754	0.1 (-0.5,0.7)	0.909
**Growth**												
**Intercept z score**	3.0 (-3.9, 10.7)	0.361	-0.0 (-1.7, 1.7)	0.965	1.2 (-2.1, 4.4)	0.478	1.2 (-1.7, 4.1)	0.415	-0.6 (-2.1, 0.9)	0.455	**1.2 (0.2, 2.3)**	**0.025**
**Slope z score**	-71.2 (-159.7, -17.3)	0.114	9.5 (-11.1, 30.2)	0.361	21.0 (-18.4, 60.4)	0.292	11.5 (-24.1, 47.0)	0.523	11.2 (-7.1, 29.6)	0.228	10.8 (-2.3, 3.9)	0.105
**Metabolic**												
**Chol, mmol/L**	-14.7 (-34.1, 4.7)	0.137	4.1 (-0.4, 8.5)	0.075	0.9 (-7.8, 9.5)	0.199	-3.2 (-10.9, 4.5)	0.412	-2.5 (-6.5, 1.4)	0.199	1.0 (-1.9, 3.9)	0.488
**LDL, mmol/L**	-13.7 (-33.3, -5.9)	0.168	3.4 (-1.2, 7.9)	0.142	0.2 (-8.5, 8.9)	0.960	-3.2 (-10.9, 4.6)	0.420	-2.1 (-6.0, 1.9)	0.306	0.4 (-2.5, 3.4)	0.783
**HDL, mmol/L**	-13.3 (-48.6, 22.0)	0.456	-3.5 (-11.6, 4.7)	0.404	-9.4 (-24.8, 6.0)	0.231	-5.9 (-19.8, 7.9)	0.398	-3.5 (-10.6, 3.6)	0.333	-0.5 (-5.8, 4.7)	0.843
**TG, mmol/L**	10.9 (-13.6, 35.5)	0.378	**5.9 (3.9, 11.6)**	**0.036**	**12.0 (1.4, 22.6)**	**0.026**	6.0 (-3.6, 15.6)	0.216	-1.3 (-3.7, 6.2)	0.616	2.4 (-1.3, 6.0)	0.198
**Insulin, mU/L**	-0.5 (-1.1, 0.8)	0.093	0.1 (-0.0, 0.2)	0.187	**0.3 (0.0, 0.5)**	**0.032**	0.2 (-0.0, 0.4)	0.106	-0.0 (-0.1, 0.1)	0.682	0.1 (-0.0, 0.2)	0.073
**Insulin resistance**	-53.6 (-118.8, 11.6)	0.106	0.3 (-15.8 16.4)	0.968	-2.9 (-32.9, 27.1)	0.847	-3.3 (-29.6, 23.1)	0.807	-5.6 (-19.2, 8.1)	0.421	-1.5 (-11.4, 8.5)	0.770
**C-peptide, ng/mL**	**-9.7 (-18.1, -1.3)**	**0.023**	0.5 (-1.6, 2.6)	0.613	3.0 (-0.8, 6.8)	0.121	2.5 (-0.9, 5.9)	0.146	-0.2 (-2.0, 1.6)	0.832	1.3, (-0.2, 2.5)	0.053
**Vit. D nmol/L**	0.4 (-0.1, 0.9)	0.139	**-0.1 (-0.2, -0.0)**	**0.035**	**-0.3 (-0.6,-0.1)**	**0.002**	**-0.2 (-0.4,-0.0)**	**0.033**	-0.1 (-0.2, 0.0)	0.269	-0.1 (-1,0)	0.177
**lnCRP**	-21.2(-45.2,-2.7)	0.081	5.3 (-0.2, 10.8)	0.060	9.1 (-1.4, 19.6)	0.090	3.8 (-5.7, 13.3)	0.431	-0.5 (-4.4, 5.4)	0.847	-0.6 (-4.2, 3.0)	0.748

Data are unstandardised β coefficients and 95% CI. Bolded values are statistically significant (p<0.05). ASMM, appendicular skeletal muscle mass; BMI, body mass index; Chol, total cholesterol; HDL, HDL high density lipoprotein; HR heart rate, LDL, low density lipoprotein; LnCRP, natural logarithm of C-reactive protein; TG, triglycerides.

**Table 3:** Predictors of physical function measures for 102 boys by linear regression adjusted for age.

Predictors	6min Walk, m	P	Heart rate 0 min, bpm	P	Heart rate 6min, bpm	P	Heart rate Δ 0–6 min bpm		Heart rate Δ 6- 7min bpm	P	Handgrip strength, kg	P
**Anthropometric**												
**Weight, kg**	**-0.8 (-1.2,-0.4)**	**<0.0001**	0.3 (-0.1, 0.1)	0.517	0.1 (-0.0, 0.3)	0.131	**0.2 (0.0, 0.3)**	**0.045**	0.1 (-0.0, 0.2)	0.163	**0.2 (0.1, 0.2)**	**<0.0001**
**Height, cm**	0.8 (-0.9, 2.4)	0.367	-0.2 (-0.5, 0.2)	0.341	-0.4 (-0.7, 0.6)	0.889	0.1 (-0.5, 0.7)	0.582	-0.1 (-0.4, 0.3)	0.761	**0.6 (0.4, 0.9)**	**<0.0001**
**BMI, kg/m^2^**	**-3.2 (-4.6, -1.8)**	**<0.0001**	-0.1 (-0.4, 0.3)	0.274	0.5 (-0.1, 1.1)	0.080	**0.6 (0.0, 1.1)**	**0.038**	0.2 (-0.1, 0.6)	0.117	**0.4 (0.2, 0.7)**	**<0.0001**
**Fat, kg**	**-1.5 (-2.2, -0.9)**	**<0.0001**	0.01 (-0.1, 0.2)	0.880	**0.3 (0.1, 0.6)**	**0.015**	**0.3 (0.1, 0.6)**	**0.012**	0.1 (-0.0, 0.3)	0.054	**0.1 (0.0, 0.3)**	**0.015**
**Fat, %**	**-2.8 (-3.9,-1.8)**	**<0.0001**	0.1 (-0.2,0.3)	0.585	**0.7 (0.2, 1.1)**	**0.003**	**0.6 (0.2, 1.0)**	**0.005**	**0.3 (0.0, 0.5)**	**0.037**	0.1 (-0.1, 0.3)	0.423
**ASMM, kg**	**-2.2 (-4.2, 0.1)**	**0.034**	**-0.4 (-0.8, 0.1)**	**0.048**	0.0 (0.0, 1.0)	0.899	0.4 (-0.3,1.1)	0.311	0.0 (-0.0,0.0)	0.767	**1.1 (0.8, 1.3)**	**<0.0001**
**ASMM, %**	**7.1 (4.4, 9.9)**	**<0.0001**	-0.4 (-1.1, 0.2)	0.214	**-2.0 (-3.1, -0.8)**	**0.001**	**-1.6 (-2.6, -0.5)**	**0.005**	**-0.8 (-1.4, -0.2)**	**0.015**	0.2 (-0.3,0.7)	0.410
**Growth**												
**Intercept z score**	-7.4 (-16.8, 1.9)	0.119	-1.3 (-3.2, 0.7)	0.207	-0.2 (-3.8, 3.5)	0.930	1.1 (-2.3, 4.5)	0.521	0.6 (-1.4, 2.5)	0.529	**2.1 (0.7, 3.6)**	**0.004**
**Slope z score**	**-107.4 (-234.3, 19.6)**	0.097	4.7 (-22.5, 31.9)	0.721	26.0 (-23.3, 75.4)	0.298	21.3 (-25.0, 57.6)	0.363	2.5 (-23.6, 28.7)	0.848	**22.7 (2.6, 42.9)**	**0.027**
**Metabolic**												
**Chol, mmol/L**	**-18.2(-33.1, -3.3)**	**0.017**	1.4 (-1.8, 4.6)	0.391	3.4 (-2.4, 9.2)	0.255	2.0 (-3.5, 7.5)	0.477	-0.7 (-2.2, 4.0)	0.553	1.3 (-1.2, 3.7)	0.299
**LDL, mmol/L**	**-18.7 (-35.4, -2.1)**	**0.028**	1.3 (-2.3, 4.9)	0.485	4.7 (-1.7, 11.3)	0.150	3.5 (-2.6, 9.6)	0.260	0.9 (-1.7, 5.2)	0.316	1.4 (-1.3,4.2)	0.298
**HDL, mmol/L**	-5.5 (-38.1, 49.2)	0.802	4.8 (-4.4, 13.9)	0.305	-5.8 (-22.4, 11.0)	0.496	-10.5 (-26.1, 5.1)	0.184	-4.7 (-13.5, 4.1)	0.295	-4.8 (-11.7 2.2)	0.175
**TG, mmol/L**	-17.6 (-39.7, 4.6)	0.120	-1.1 (-5.9, 3.6)	0.622	0.8 (-7.9, 9.5)	0.885	2.0 (-6.1, 10.1)	0.629	0.3 (-4.3, 4.8)	0.901	3.4 (-0.1, 6.9)	0.060
**Insulin, mU/L**	-0.4 (-1.0, 0.2)	0.166	0.1 (0.0, 0.2)	0.171	**0.3 (0.1, 0.6)**	**0.005**	**0.2 (0.0, 0.5)**	**0.031**	0.1 (-0.1, 0.2)	0.183	0.0 (-0.1, 0.1)	0.570
**Insulin resistance**	-24.5 (-50.5, 99.6)	0.729	14.7 (-1.0 30.4)	0.066	5.8 (-23.4, 35.1)	0.693	-8.9 (-36.0, 18.3)	0.518	-4.0 (-19.4, 11.5)	0.616	-4.9 (-17.0, 7.3)	0.427
**C-peptide, ng/mL**	**-11.6 (-20.4, -2.8)**	**0.010**	-0.4 (-2.3, 1.5)	0.685	3.3 (-0.2, 6.8)	0.061	**3.7 (0.5, 6.9)**	**0.023**	1.0 (-0.8, 2.9)	0.267	1.3, (-0.1, 2.8)	0.077
**Vit. D nmol/L**	**0.6 (0.1, 1.2)**	**0.026**	**-0.1 (-0.3, -0.0)**	**0.023**	**-0.3 (-0.5, -0.1)**	**0.011**	**-0.1 (-0.3,0.1)**	**0.177**	-0.1 (-0.2, 0.1)	0.311	0.0 (-0.1,0.1)	0.963
**lnCRP**	-17.1 (-51.9, -17.7)-	0.332	2.8 (-4.6, 10.1	0.457	5.2 (-8.2, 18.6)	0.442	2.5 (-10.2, 15.1)	0.700	-1.2 (-8.3, 5.9)	0.736	-2.4 (-8.0, 3.2)	0.392

Data are unstandardised β coefficients and 95% CI. Bolded values are statistically significant (p<0.05). ASMM, appendicular skeletal muscle mass; BMI, body mass index; Chol, total cholesterol; HDL, HDL high density lipoprotein; HR heart rate, LDL, low density lipoprotein; LnCRP, natural logarithm of C-reactive protein; TG. triglycerides.

Stepwise regression analyses performed for girls (Table 4) and boys (Table 5) separately. For both sexes, age and the proportion of the body mass which was ASMM were independently predictive of distance walked. Controlling for %ASMM, for one year of age increase the mean distance walked was 34 (95%CI 15, 53) m less in girls and 59 (95%CI 36, 84) m less in boys. Resting heart rate was inversely related to %ASMM and directly associated with triglyceride concentration in girls (Table 4) while, in boys (Table 5), resting heart rate was inversely related to both vitamin D concentration and ASMM. The best predictors of a lower heart rate after 6 minutes of walking were, for girls, age and vitamin D concentration and, for boys, age and %ASMM. A greater increase in heart rate over the six-minute walk was associated with lower age in both sexes and independently with lower vitamin D concentration in girls (Table 4) and reduced %ASMM in boys (Table 5). The fall in heart rate in the minute following the walk was greater with increased %ASMM and age in boys (Table 5) but was not associated with any of the measured predictors in girls (Table 4). Reduced body fat and increased ASMM were independently predictive of higher handgrip strength in both girls and boys.

**Table 4:** Multiple regression analyses for prediction of physical function for 98 girls.

	Unstandardised			Standardised	P value	R^2^_adj_*
	β	Std Error	95% CI	β		
**Distance walked in 6 minutes, m**						
**Age, y**	-34.3	9.5	(-53.3, -15.3)	-0.336	<0.0001	0.087
**ASMM%**	6.3	2.1	(2.2, 10.4)	0.284	0.001	0.159
**Heart rate, bpm, 0 min**						
**ASMM%**	-1.1	0.5	(-2.1, -0.2)	-2.26	0.024	0.046
**TG, mmol/L**	5.8	2.6	(0.6, 11.1)	0.217	0.030	0.084
**Heart rate, bpm, 6 min**						
**Age, y**	-13.5	4.2	(-21.9, -5.1)	-0.301	0.002	0.079
**Vitamin D, nmol/L**	-0.35	0.11	(-0.57, -0.13)	-0.296	0.002	0.159
**Heart rate, bpm, Δ 0–6 min**						
**Age, y**	-11.6	3.9	(-19.3, -3.9)	-0.290	0.004	0.073
**Vitamin D, nmol/L**	-0.22	0.10	(-0.42, -0.02)	-0.210	0.033	0.108
**Heart rate, bpm, Δ 6- 7min**						
**No significant predictors**						
**Handgrip strength, kg**						
**ASMM, kg**	1.40	0.22	(0.97, 1.84)	0.937	<0.0001	0.320
**Fat, kg**	-0.23	0.08	(-0.38, -0.08)	-0.447	0.001	0.368

Data are unstandardised and standardised β coefficients and 95% CI. ASMM, appendicular skeletal muscle mass; TG, triglycerides. *R^2^_adj_, adjusted R^2^ values for sequential addition of variables.

**Table 5:** Multiple regression analyses for prediction of physical function for 102 boys.

	Unstandardised coefficients			Standardised	P value	R^2^_adj_*
	β	Std Error	95% CI	β		
**Distance walked in 6 minutes, m**						
**ASMM%**	7.1	1.4	(4.4, 9.9)	0.415	<0.0001	0.172
**Age, y**	-58.7	12.1	(-83.5, -36.0)	-0.407	<0.0001	0.332
**Heart rate, bpm, 0 min**						
**Vitamin D, nmol/L**	-0.15	0.06	(-0.26, -0.04)	-0.256	0.009	0.042
**ASMM, kg**	-0.47	0.20	(-0.86, -0.07)	-2.355	0.021	0.084
**Heart rate, bpm, 6 min**						
**Age, y**	-19.0	4.9	(-28.7, -9.3)	-0.347	<0.0001	0.107
**ASMM%**	-1.96	0.57	(-3.10, -0.83)	-0.308	0.001	0.195
**Heart rate, bpm, Δ 0–6 min**						
**Age, y**	-18.5	4.7	(-27.7, -9.2)	-0.358	<0.0001	0.116
**ASMM%**	-1.55	0.54	(-2.63, -0.48)	-0.259	0.005	0.175
**Heart rate, bpm, Δ 6- 7min**						
**Age, y**	-10.5	2.6	(-15.7, -5.2)	-0.361	<0.0001	0.118
**ASMM%**	-0.77	0.31	(-1.38, -0.15)	-0.226	0.015	0.161
**Handgrip strength, kg**						
**ASMM, kg**	1.65	0.16	(1.34, 1.96)	0.993	<0.0001	0.471
**Fat, kg**	-0.26	0.06	(-0.37, -0.14)	-0.425	<0.0001	0.556

Data are unstandardised and standardised β coefficients and 95% CI. ASMM, appendicular skeletal muscle mass. *R^2^_adj_, adjusted R^2^ values for sequential addition of variables.

## Discussion

Our aim was to explore relationships between physical function, body composition and metabolic health in Pacific Island youth. In particular, in line with the hypothesis, we found for both girls and boys, after controlling for age, the proportion of ASMM positively predicted distance walked in 6 min and a lower heart rate after 6 min of walking. Increasing age was associated with significantly reduced walking distance, independently of percent ASMM. Handgrip strength, as another marker of physical function, was independently predicted by whole-body fat mass and ASMM and these variables acted in opposite directions; increasing fat mass predicted reduced handgrip strength. A lower heart rate after 6 min of walking was predicted also by higher plasma vitamin D concentrations.

Our results confirm those found in a recent study with 165 youth aged 14 years showing that higher physical fitness, as assessed by peak oxygen uptake during exercise, can be explained by a higher proportion of skeletal muscle mass, as estimated by bioimpedance [25]. Explanation of the decrease in distance walked with age could include an interaction of decreasing intrinsic motivation [26] with increasing body and fat mass in these rapidly growing and maturing youth. The youth in the present substudy were measured on average one year after the main study [17]. In this time those boys and girls gained on average 8 and 4 kg of weight and 5 and 2 cm in height, respectively. Compared to height-related reference centiles in healthy children living in Hong Kong [27], the average boy and girl in this study walked less distance than the 10^th^ sex-specific centile (less than the lowest 10^th^ percentile comparisons).

Associations between vitamin D and physical fitness have been investigated by others. In a large study of male university students, Park et al. [27] showed that vitamin D concentrations were directly correlated with maximum oxygen uptake measurements. This positive correlation was also seen in an equally large study of black and white youth in the south eastern United States aged 14–18 who underwent submaximal oxygen uptake testing [28] and in males of similar age range who underwent fitness testing [29]. These relationships may result from increased time spent outdoors. Reduced body fat or BMI may also play a role given the negative correlations observed between vitamin D and body fatness [28–30] that may be a consequence of vitamin D sequestration in adipose tissue [31].

We are not aware of any studies that have looked at muscle mass and functional measures of physical fitness apart from peak oxygen uptake[25]. Few studies have “tracked” relative physical function across childhood and youth [32]. Poor tracking of diet and fitness from adolescence to young adulthood has previously been reported [33]. One study has shown that a larger increment in body fatness (measured by skinfolds) between childhood to adolescence was associated with reduced physical fitness (9 minute run) [28].

Strengths of this report include the measurement of whole body composition, characterisation of the growth trajectory over the preceding years and comprehensive measures of metabolic health. The study is limited in that there were few measures of physical fitness but compliance with the testing was excellent (99%) and the tests were simple to perform. The sample size was relatively small and the number of variables considered relatively large so the number of variables in any model had to be carefully chosen. Variation in testing was minimised as one research assistant (DR) undertook all the walk testing and this person had established a rapport with the youth and their families as he also explained the study and gained the informed consent when he visited the home of the family, days before the test was undertaken. In addition, as part of an ongoing prospective study, this analysis will help inform future research questions and explain other outcomes such as onset of diabetes.

It is recognised that maximal or peak values of oxygen uptake measured at high intensity work rates are considered the gold standard of cardiorespiratory fitness testing [34] but this requires compliance, skilled expertise and expensive equipment for the measurement of gas exchange. While the 6MWT is not a gold standard measure of fitness it has wide acceptance and utility, particularly for non-athletes and children [35] as a pragmatic measure of functional capacity. The measurement of recovery heart rates following the 6MWT is a novel measure of fitness. The use of DXA to precisely characterise ASMM and body fat strengthened the ability to explore relationships between metabolic health, body composition and functional capacity.

In general high body mass index is a predictor of metabolic risk but for the same BMI as other ethnic groups Pacific people have more muscle and less fat [8]. We have shown in this unique population that at a critical period of growth and development there are associations between physical function, body composition and metabolic health. In particular, improved metabolic health with increased ASMM suggests that interventions earlier in the lifecourse could be beneficial. We support suggestions from other teams that interventions that focus on both strength [5,36] and aerobic activities [6] could have a beneficial effect on future health and add that the mediator may be the quantity and quality of ASMM. In New Zealand, Pacific people have a disproportionately higher prevalence of type 2 diabetes at younger ages than other ethnic groups[13]. An emphasis on both muscular power and aerobic activity in childhood may help reduce insulin resistance [36]

A negative, albeit cross-sectional, association of age and ASMM with distance walked is of interest and should be explored further. Understanding of the interrelationships between physical growth, body composition, physical function and metabolic health should be further investigated in the context of other influences such as food security, opportunities for physical activity, cultural expectations and the food and activity environment.
